# Value of combined detection of PCA, ANCA, ASCA, AGA and ANA in early diagnosis of Gastrointestinal Diseases

**DOI:** 10.12669/pjms.38.1.4682

**Published:** 2022

**Authors:** Xin Liu, Deli Guo, Xiaoyan Li, Yaping Guo, Wenjie Song

**Affiliations:** 1 Xin Liu, Department of Clinical Laboratory, Baoding NO.1 Central Hospital, Baoding, 071000, Hebei, China; 2 Deli Guo, Department of Orthopedics, Baoding NO.1 Central Hospital, Baoding, 071000, Hebei, China; 3 Xiaoyan Li, Department of Clinical Laboratory, Baoding NO.1 Central Hospital, Baoding, 071000, Hebei, China; 4 Yaping Guo, Department of Clinical Laboratory, Baoding NO.1 Central Hospital, Baoding, 071000, Hebei, China; 5 Wenjie Song, Department of Clinical Laboratory, Baoding NO.1 Central Hospital, Baoding, 071000, Hebei, China

**Keywords:** Anti-parietal cell antibody, Anti-neutrophil cytoplasmic antibody, Anti-Saccharomyces cerevisiae antibody, Anti-gliadin antibody, Anti-nuclear antibody

## Abstract

**Objectives:**

To investigate the positive detection rate and clinical application value of anti-parietal cell antibody (PCA), anti-neutrophil cytoplasmic antibody (ANCA), anti-Saccharomyces cerevisiae antibody (ASCA), anti-gliadin antibody (AGA) and anti-nuclear antibody (ANA) in patients visiting the Department of Gastroenterology.

**Methods:**

From January 2015 to June 2018, 1,083 patients receiving detection of PCA and other autoantibodies were selected from the Department of Gastroenterology of Baoding First Central Hospital. The positive detection rate of autoantibodies was statistically analyzed. The enumeration data were expressed as rate or constituent ratio, and the rates were compared between groups using the x^2^ test.

**Results:**

Among the 1,083 patients, the positive detection rate of ANA, ASCA, AGA, PCA and ANCA was 33.52%, 16.71%, 15.51%, 13.66% and 7.66%, respectively. The total positive detection rate was 62.8% (n = 680).

**Conclusion:**

The population with abdominal distension, chronic abdominal pain, diarrhea and other digestive system symptoms should be timely treated with combined detection of PCA, ANCA, ASCA, AGA and ANA, which is of important clinical application value for early diagnosis of gastrointestinal diseases and prevention of missed diagnosis and misdiagnosis.

## INTRODUCTION

Abdominal distension, chronic abdominal pain and diarrhea are common symptoms of patients visiting the Department of Gastroenterology, which are not specific and can be caused by multiple diseases. The factors causing gastrointestinal diseases are numerous, and the etiology is complex. The common ones include infection, physicochemical factors, metabolic and absorption disorders, autoimmunity, etc., among which autoimmune factors play an important role.^1^ Autoantibodies refer to the antibodies against the tissues, organs, cells and cell components of the body, which are immunoglobulins produced by the immune response to the body’s own components due to immune dysfunction. With the development of detection technologies and the development and application of various autoantibodies, the incidences of autoimmune gastrointestinal diseases such as inflammatory bowel disease (IBD), type A atrophic gastritis (AIG) and celiac disease have been increasing in recent years.^2^-^4^ Because of atypical early symptoms and similar clinical manifestations, their diagnosis mostly depends on endoscopy and pathology, and pathological development is a slow and gradual process, always resulting in diagnosis many years after the symptoms appear and very severe outcomes in many cases. Therefore, it is of great significance for the precise diagnosis and treatment of diseases to accurately determine the types of diseases in the early stage by serological autoantibody detection. In this paper, the combined detection results of PCA, ANCA, ASCA, AGA and ANA in the Department of Gastroenterology of our hospital were analyzed and discussed.

## METHODS

From January 2015 to June 2018, 1,083 patients receiving detection of PCA, ANCA, ASCA, AGA and ANA were selected from the Outpatient and Inpatient Department of Gastroenterology of Baoding First Central Hospital. Exclusion criteria included: patients with liver diseases; patients with primary hematological diseases; and patients with mental illness.

### Ethical Approval:

The study was approved by the Institutional Ethics Committee of Baoding No.1 Central Hospital on March 01, 2021(No.:[2021-011]), and written informed consent was obtained from all participants

Equipment and reagents: BX43 fluorescence microscope was from Olympus (Japan), and MULTSKAN FC microplate reader was from ThermoFisher Instrument Co., Ltd. (Shanghai). Detection reagents for ANA and ANCA were from EUROIMMUN Medical Diagnostics Co., Ltd., and detection reagents for ACSA, AGA and PCA were from Guangzhou Kangrun Biotechnology Co., Ltd.

ANA and ANCA were detected using indirect immunofluorescence, and PCA, ASCA and AGA were detected by Western blot. The operation was strictly in accordance with the instructions. For the subjects, present situation investigation and retrospective analysis were mainly used.

### Statistical Analysis:

Statistical analysis was carried out using SPSS19.0. The enumeration data were expressed as rate or constituent ratio, and the rates were compared between groups using the x^2^ test. *P* < 0.05 was considered as statistically significant.

## RESULTS

Among the 1,083 patients, 680 patients showed five positive antibodies, with the total positive detection rate of 62.8%. The positive detection rate of ANA was the highest (33.52%). The positive detection rate of each antibody is shown in Table-I.

**Table-I:** Positive detection rate of ANA, ASCA, AGA, PCA and ANCA.

Item	N	Positive detection rate, %
ANA	363	33.52
ASCA	181	16.71
AGA	168	15.51
PCA	148	13.66
ANCA	83	7.66

A total of 480 patients presented positive PCA, ASCA, AGA and ANCA, with the positive detection rate of 44.32%. Among them, ANA was positive in 163 patients, accounting for 33.96%. ASCA, AGA and ANCA were negative in 603 patients, among which ANA was positive in 200 patients, accounting for 33.167%. No statistically significant differences were found in the positive detection rate of ANA between the autoantibody-positive group and the autoantibody-negative group, as seen in Table-II.

**Table-II:** Combined detection results of ANA between autoantibody-positive group and autoantibody-negative group.

	Positive number of ANA	Total number	χ^2^	P
Autoantibody-positive group	163	480	0.08	> 0.05
Autoantibody-negative group	200	603		

## DISCUSSION

The positive detection rate of ANA and other autoantibodies was 62.8%, suggesting that autoimmune factors account for a high proportion in gastrointestinal diseases. Previously, it was reported that the prevalence rate of gastrointestinal autoimmune diseases was very low, and some were even rare diseases.^5^ In this study, it was found that 680 of 1,083 patients in the Department of Gastroenterology had positive ANA and other autoantibodies. The causes may be analyzed as follows:

1. Autoimmunity plays an important role in the occurrence and development of diseases;
2. Because the early symptoms of gastrointestinal diseases are not typical and pathology is a process of slow progression, many gastrointestinal AID are not timely diagnosed, resulting in missed diagnosis and misdiagnosis;
3. PCA and other autoantibodies also have a certain positive rate in non-AID diseases. Therefore, PCA and other autoantibodies have important application value in the diagnosis and differential diagnosis of gastrointestinal diseases.

In this study, it was found that the positive detection rate of ANA was 33.52% in the patients visiting the Department of Gastroenterology, which was lower than that in our previous study based on a large sample size.^6^ ANA is the general term of autoantibodies targeting various components of eukaryotic cells as target antigens.^7^ ANA acts on mitotic cell cycle, resulting in production disorders of DNA and proteins and blocked metabolism, so it has strong damage to the tissues with exuberant metabolism. ANA detection is a classic screening test for AID. There is high titer of ANA in the serum of AID patients. In recent years, a study^8^ has found that ANA exists in the serum of patients before clinical symptoms appear, which is predictive for diseases. Gastrointestinal vasculitis caused by systemic lupus erythematosus is the most common cause of gastrointestinal involvement.^9^ The pathogenesis includes the deposition of immune complexes and complements of small vessels in the mesentery and intestinal wall, accompanied by inflammatory cell infiltration, and thrombosis in small vessels, which results in thickening and occlusion of the wall of small mesenteric artery, causing intestinal mucosal edema, intestinal ischemia, ulcers, bleeding intestinal obstruction, perforation, etc.^10^ One third of the patients with positive ANCA, PCA, ASCA and AGA were accompanied with positive ANA. Whether these patients have the possibility of overlap syndrome or specific markers of certain AID involving the gastrointestinal tract remains to be further explored.

The positive detection rate of ASCA was 16.71%. The main target antigen of ASCA is phosphopeptide mannan on the cell wall of Saccharomyces cerevisiae (homologous with cell wall component of intestinal flora). ASCA is involved in the pathogenesis of Crohn’s disease (CD), which is a highly specific serum marker for CD patients.^11^ The target antigens of ANCA are various components in the cytoplasm of neutrophils and monocytes.^12^ Protease 3 and myeloperoxidase are considered to be the target antigens of vasculitis. In IBD, the target antigen corresponding to pANCA is histone 1, and pANCA is considered to be an autoantibody induced by cross reaction with intestinal bacterial antigens.^13^ pANCA can be detected in 60-70% patients with ulcerative colitis (UC). IBD is still a rare disease in China, but its incidence has been increasing rapidly in recent 20 years. It has been shown^14^ that the incidence of UC (1.64/10 million) in China is higher than that of CD (0.13/10 million), while the missed diagnosis rate of CD is 60.9%, which is nearly 1 time higher than that of UC (32.1%). However, our study revealed that the positive detection rate of ASCA was 16.77%, which was higher than that of ANCA (7.66%), confirming that many patients with CD may not be diagnosed, and the missed diagnosis is not optimistic.

The target antigens of AGA is a group of proteins rich in proline and glutamine, which are the main toxic components of gluten. These proteins can not be digested in the gastrointestinal tract. Through connexin, the cytoskeleton is reconstructed and the tight junction is destroyed, leading to increased permeability of the small intestinal mucosa. The toxic effect of gliadin also reduces the fibroactin of small intestinal epithelial cells, inhibits the growth of epithelial cells, and induce apoptosis.^15^ AGA is a serological marker for celiac disease. The prevalence rate of celiac disease is much higher than expected, and only 10-15% of the patients are diagnosed.^16^ This investigation found that its positive detection rate in patients in the Department of Gastroenterology was 15.51%, which was far higher than its prevalence rate. The prevalence rate of celiac disease is higher in the West.^17^ With the development of society, the change of dietary structure and the improvement of laboratory detection technology, the prevalence rate of celiac disease in China is increasing.

The target antigens of PCA are H^+^/K^+^-ATPase and gastrin receptor on the surface of gastric parietal cells. After PCA formation, the mucosa of the gastric body and fundus where gastric parietal cells are located is damaged and atrophied, the glands are decreased, and then the function is reduced, leading to dyspepsia symptoms such as abdominal distension, and forming chronic atrophic gastritis after a long term. Chronic atrophic gastritis is a common clinical disease in China, without gold standard for its diagnosis.^18^ PCA-positive is the characteristic of type A atrophic gastritis, which mainly causes atrophy of the gastric body. Helicobacter pylori infection can lead to chronic non-atrophic gastritis and atrophic gastritis, mostly presenting multifocal gastric mucosal atrophy, which is called type B atrophic gastritis. The rate of Helicobacter pylori infection is high in China. In the past, it was believed that atrophic gastritis in China was mainly type B atrophic gastritis, and type A atrophic gastritis was rare.^19^ Our study found that the positive detection rate of PCA was 13.66%, which was much higher than the prevalence rate. It has been reported that Helicobacter pylori can activate self-reactive T lymphocytes and trigger autoimmune mechanism through the molecular simulation mechanism at the level of T lymphocytes, thus leading to type A atrophic gastritis.^20^ Therefore, the prevalence rate of type A atrophic gastritis is likely to be underestimated, mainly caused by the high incidence of Helicobacter pylori infection in China, the lack of PCA detection and the gradual process of pathological changes. Consequently, PCA detection can be used as a routine screening of gastritis, which is of great significance for clarifying the etiology and early prevention and treatment.

IBD, celiac disease, atrophic gastritis and other autoimmune gastrointestinal diseases have been increasing in recent years in China, but their incidences are still low. Without gold standard, they are mainly comprehensively diagnosed based on clinical manifestations, laboratory indexes, endoscopic and pathological results. The early clinical manifestations of these autoimmune gastrointestinal diseases are similar, and the pathological progression is a gradual process, which leads to the high rate of missed diagnosis and misdiagnosis. However, in our study, it was revealed that the positive detection rates of specific serological markers for these diseases were much higher than their prevalence rates, proving that these markers already exist in the serum of patients before diagnosis, and can be used as a good detection method, which has important value for the early diagnosis of the diseases. Therefore, the detection rate of these diseases can be improved by expanding the detection range of autoantibodies and clinical combined detection.

### Limitations of this study:

Our sample size is not large enough. In future studies, countermeasures are being taken to further accumulate patient information, increase the sample size, extend the follow-up time, and carry out more stringent experimental design, so as to make up for the above shortcomings and be more conducive to guiding clinical practice.

## CONCLUSION

The population with abdominal distension, chronic abdominal pain, diarrhea and other digestive system symptoms should be timely treated with combined detection of PCA, ANCA, ASCA, AGA and ANA, which is of important clinical application value for early diagnosis of gastrointestinal diseases and prevention of missed diagnosis and misdiagnosis.

### Authors’ Contributions:

**XL & DG:** Designed this study and prepared this manuscript, and are responsible and accountable for the accuracy or integrity of the work.

**XL** and **YG:** Collected and analyzed clinical data.

**WS:** Significantly revised this manuscript.
